# Supplementary material for: Predicting visual function by interpreting a neuronal wiring diagram
Source: Nature. 2024 Oct 2;634(8032):113–23. doi: 10.1038/s41586-024-07953-5 (PMC11446822; doi:10.1038/s41586-024-07953-5)
Supplement: Supplementary file 6 — CRF and ERF predictions for individual TmY4 and TmY9 cells. Analogous to Supplementary Data 3, but for TmY target types. Shown are the top four monosynaptic pathways, the strongest pathway passing through each of the top ten intermediary types (ranking from Extended Data Fig. 7), and the trisynaptic pathway Tm1–TmY–Dm3–TmY (see the section entitled Prediction of spatial normalization). [file 41586_2024_7953_MOESM6_ESM.zip › DataS4/TmY9q/720575940625520446.pdf]

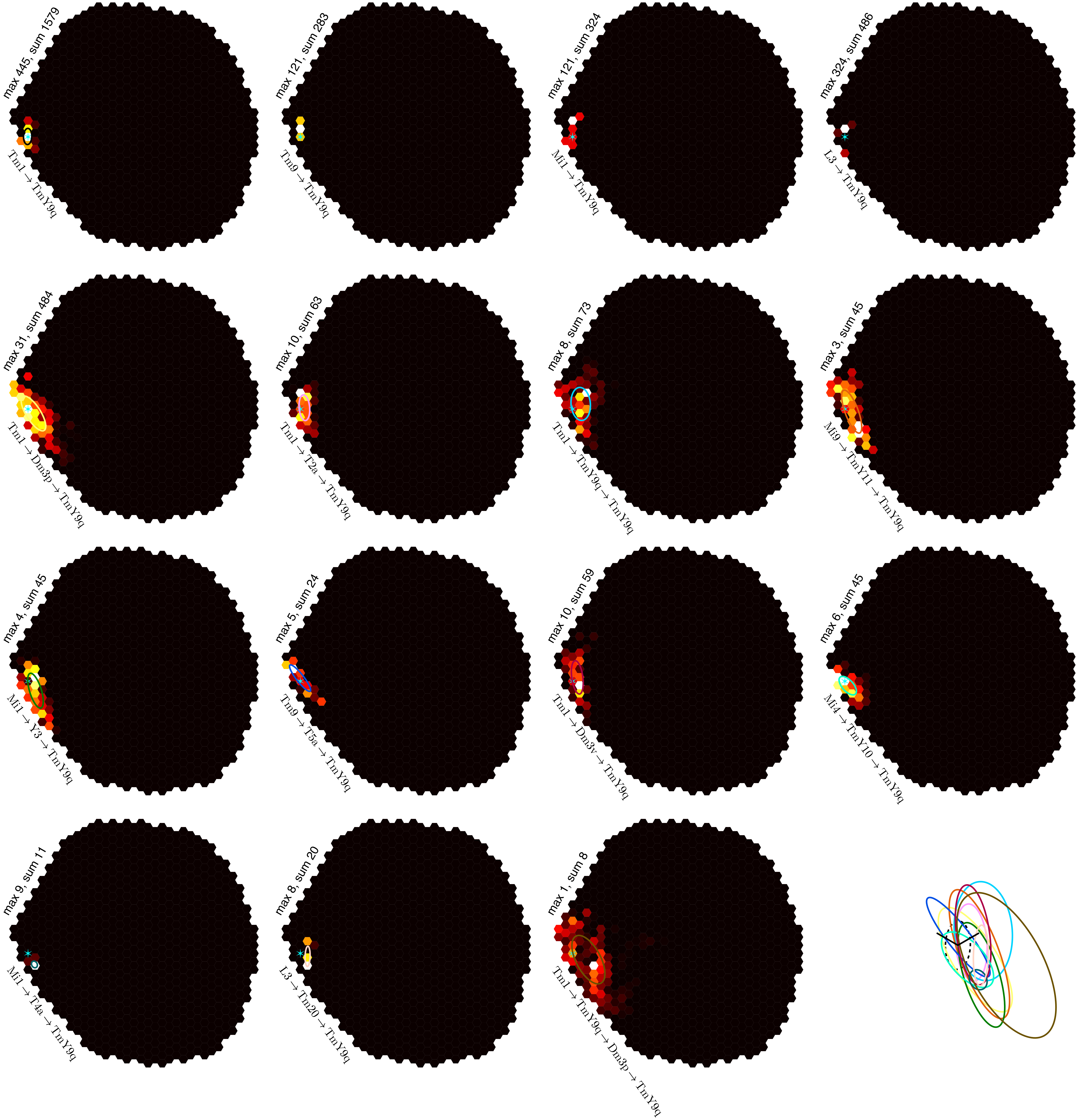
max 445, sum 1579
Tm1 → TmY9q
max 121, sum 283
Tm9 → TmY9q
max 121, sum 324
Mi1 → TmY9q
max 324, sum 486
L3 → TmY9q
max 31, sum 484
Tm1 → Dm3p → TmY9q
max 10, sum 63
Tm1 → T2a → TmY9q
max 8, sum 73
Tm1 → TmY9q → TmY9q
max 3, sum 45
Mi9 → TmY11 → TmY9q
max 4, sum 45
Mi1 → Y3 → TmY9q
max 5, sum 24
Tm9 → T5a → TmY9q
max 10, sum 59
Tm1 → Dm3v → TmY9q
max 6, sum 45
Mi4 → TmY10 → TmY9q
max 9, sum 11
Mi1 → T4a → TmY9q
max 8, sum 20
L3 → Tm20 → TmY9q
max 1, sum 8
Tm1 → TmY9q → Dm3p → TmY9q
